# Cloacal Exstrophy Repair with Primary Closure of Bladder Exstrophy: A Case Report and Review of Literature

**DOI:** 10.1155/2016/8538935

**Published:** 2016-05-23

**Authors:** George Bethell, Navroop Johal, Peter Cuckow

**Affiliations:** ^1^Institute of Child Health, University of Liverpool, Liverpool L69 3BX, UK; ^2^Great Ormond Street Children's NHS Trust, Great Ormond Street, London WC1N 3JH, UK

## Abstract

Cloacal exstrophy is the most complex congenital, ventral, abdominal wall defect. Traditionally surgery consists of a staged approach to repair which takes place on many separate theatre visits. In this case a primary approach was undertaken resulting in a relatively short inpatient stay and a reduced risk from multiple surgical procedures under general anaesthesia.

## 1. Introduction

Cloacal exstrophy (CE) is the most complex congenital, ventral, abdominal wall defect with an incidence of less than 1 in 200,000 live births [1]. Conventionally surgical repair consisted of a multiple staged approach; however here we describe a primary approach in a male neonate undertaken by two surgical specialties.

## 2. Case Report

A male neonate was born at 39 weeks' gestation by normal vaginal delivery to nonconsanguineous parents. He was diagnosed with CE. Antenatally, bladder exstrophy was suspected at week 21 due to nonvisualisation of the bladder on foetal ultrasonography and the decision was made to continue the pregnancy. The remainder of the gestation and labour were unremarkable and he has a healthy brother aged 3 years old.

**Figure 1:**
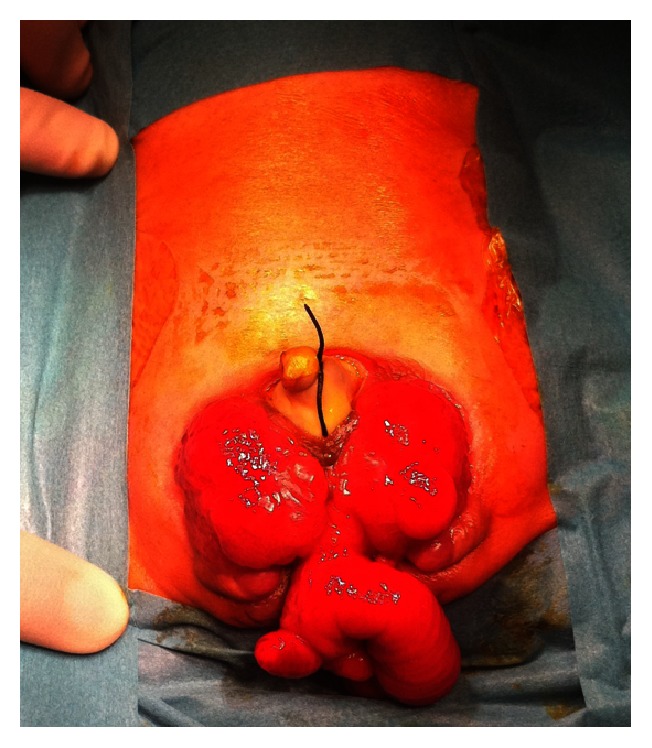
The appearance of the patient's abdomen before surgery at nine days old. The omphalocele is tied, hemibladder plates are extrophied along with caecum. The hemiphalli are inferior to the hemibladder plates.

Immediate management consisted of dressing the defect to minimalize fluid losses and the risk of infection. He was then transferred, on day 1 of life, from a district general hospital to a tertiary pediatric unit, where intravenous antibiotics were given. Neurological examination and spinal ultrasound scan did not find evidence of myelocystocele. On full examination we found an imperforate anus, bifid scrotum, and pubic symphysis diastasis. The small omphalocele was tied as shown in Figure 1.

On day 9 of life the patient was taken to theatre where primary closure of cloacal exstrophy with bilateral oblique pelvic innominate osteotomies was undertaken. Firstly the orthopaedic team performed pelvic osteotomies, followed by the pediatric urology team closing the exstrophied caecum with anastomosis to the hind gut, creating an end colostomy, and closing the omphalocele with excision of the appendices. The bladder plates were then mobilised and sutured together dorsally and ventrally. The abdominal wall was then closed ventrally with a catheter placed in the neck of the bladder and a drain placed in each hemibladder wall. Finally orthopaedics applied a spica cast. Overall theatre time was five and a half hours.

**Figure 2:**
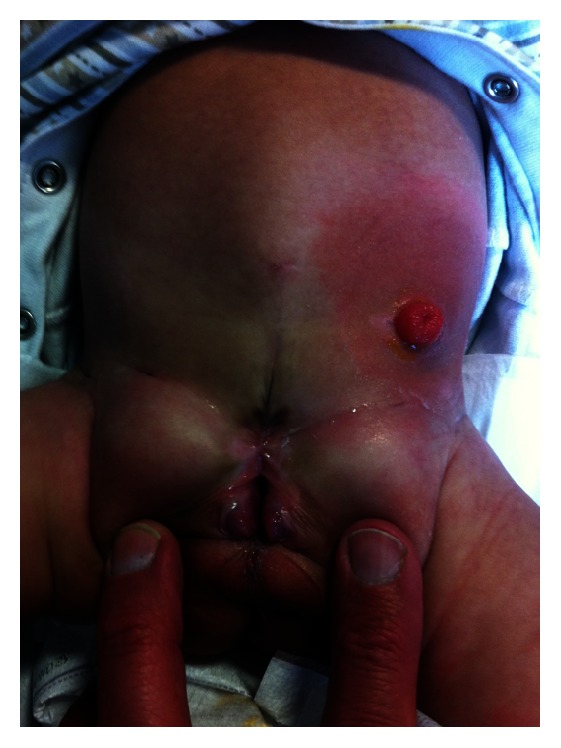
The appearance of the patient's abdomen after surgery in clinic at 8 weeks old. The end colostomy is visible along with the opening of the bladder neck and the hemiphalli.

The patient was started on total parenteral nutrition on his return to the urology high dependency unit; urine output was monitored closely along with the other vital signs. The catheters were removed; he was discharged home three weeks following surgery and then seen in clinic when he was eight weeks old (Figure 2). He developed no known complications. Future surgical treatment will involve a bladder neck reconstruction with epispadias repair and a pull through procedure if the external anal sphincter is found to be functioning with electrical stimulation.

## 3. Discussion

CE has an incidence of between 1 in 200,000 and 1 in 400,000 live births; there is no known genetic component to the aetiology. In normal embryology, the cloaca is a common tract which at around week four separates, by growth of the urogenital membrane, into the anorectal canal and the urogenital sinus. The urogenital membrane then differentiates into the perineal body.

At around the same time, the cloacal membrane which separates the cloaca from the amniotic fluid is invaded by lateral mesodermal folds. This forms the ventral abdominal wall. CE occurs when this invasion of mesoderm fails resulting in rupture of a weakened cloacal membrane before 4 weeks' gestation. The urogenital membrane also fails to develop [1]. Typical features of this defect are two exstrophied bladder plates separated medially by an exstrophied caecal plate, an omphalocele, intussusception, and herniation of the ileum, hemiphalli, imperforate anus with a variable length of shortened hindgut, and diastasis of the pubic symphysis [2]. See Figure 1.

In western countries the majority of cases of CE are diagnosed in the prenatal period following foetal ultrasound scanning [1]. Due to the complexity of the defect and lack of long term data, prenatal counselling is offered to most mothers with many opting for termination [3]. The first case of a patient surviving CE beyond the neonatal period was published in 1960 [4]. Surgical, medical, and nutritional advances over the following decades have brought present day survival to between 83 and 100% in the western world. This includes the use of intensive care units after surgery, improved nutritional care including total parental nutrition, and developments in antibiotics [1, 5]. Traditionally surgery is undertaken as a multiple staged approach. In the first procedure the intestine is dissected from the hemibladder plates and a stoma is formed. The bladder plates are then joined dorsally. The next procedure involves closure of the hemibladders ventrally but this can require multiple procedures to gain full closure. The patient then returns to theatre at around 6 months of age for closure of the abdominal wall, which can also require multiple attempts to gain full closure. The pelvic osteotomies would also require a separate visit to theatre under orthopaedics [6]. Outcomes from this approach have been undesirable with reports of only 5 out of 10 and 1 out of 10 of the surviving fourteen patients achieving urinary and faecal continence, respectively [7].

In this case, surgery was undertaken as a primary repair under the care of two surgical specialties resulting in full bladder and abdominal wall closure within the first few days of life. Indications for primary closure are that the patient is haemodynamically stable, there is adequate pulmonary function to cope with the increased abdominal pressure, the omphalocele is small, and the pubic diastasis is small [6]. This method was first described in 1999 [2]. So far there is no long term data on this approach; however short term observations are that the patient leaves hospital faster and avoids the burden of multiple operations in the early stages of life. The risks of multiple procedures in an infant include infection, tracheal stenosis, hypothermia, electrolyte imbalance, coagulopathy, and death [8].

CE represents a significant reconstructive surgical challenge with the aims of preventing upper urinary tract damage, achieving urinary and faecal continence, and leaving an acceptable cosmetic appearance. As more long term data is collected it is anticipated that this will be shown to be the desired surgical approach in the properly selected patient. Patients with large omphaloceles, open myelomeningoceles, and other life threatening issues need to have these problems addressed initially.
